# Effect of anti-podoplanin antibody administration during lipopolysaccharide-induced lung injury in mice

**DOI:** 10.1136/bmjresp-2017-000257

**Published:** 2017-11-08

**Authors:** Sian Lax, Julie Rayes, David R Thickett, Steve P Watson

**Affiliations:** 1 Institute of Cardiovascular Science, College of Medical and Dental Sciences, University of Birmingham, Birmingham, UK; 2 Institute of Inflammation and Ageing, University of Birmingham Research Labs, QE Hospital, Birmingham, UK

**Keywords:** ards, macrophage biology

## Abstract

**Introduction:**

Acute respiratory distress syndrome (ARDS) is a devastating pulmonary condition in the critically ill patient. A therapeutic intervention is yet to be found that can prevent progression to ARDS. We recently demonstrated that the interaction between podoplanin expressed on inflammatory alveolar macrophages (iAMs) and its endogenous ligand, platelet C-type lectin-like 2 (CLEC-2), protects against exaggerated lung inflammation during a mouse model of ARDS. In this study, we aim to investigate the therapeutic use of a crosslinking/activating anti-podoplanin antibody (α-PDPN, clone 8.1.1) during lipopolysaccharide (LPS)-induced lung inflammation in mice.

**Methods:**

Intravenous administration of α-PDPN was performed 6 hours after intratracheal LPS in wildtype, C57Bl/6 mice. Lung function decline was measured by pulse oximetry as well as markers of local inflammation including bronchoalveolar lavage neutrophilia and cytokine/chemokine expression. In parallel, alveolar macrophages were isolated and cultured in vitro from haematopoietic-specific podoplanin-deficient mice (Pdpn^fl/fl^VAV1cre^+^) and floxed-only controls treated with or without LPS in the presence or absence of α-PDPN.

**Results:**

Lung function decline as well as alveolar neutrophil recruitment was significantly decreased in mice treated with the crosslinking/activating α-PDPN in vivo. Furthermore, we demonstrate that, in vitro, activation of podoplanin on iAMs regulates their secretion of proinflammatory cytokines and chemokines.

**Conclusions:**

These data confirm the importance of the CLEC-2–podoplanin pathway during intratracheal (IT)-LPS and demonstrate the beneficial effect of targeting podoplanin during IT-LPS in mice possibly via modulation of local cytokine/chemokine expression. Moreover, these data suggest that podoplanin-targeted therapies may have a beneficial effect in patients at risk of developing ARDS.

**Key messages**

- Administration of an anti-podoplanin antibody reduces arterial oxygen decline and alveolar neutrophilia during intratracheal lipopolysaccharide in mice.
- Podoplanin expression on inflammatory alveolar macrophages regulates their secretion of cytokine/chemokines in vitro.
- Targeting podoplanin may be beneficial in patients at risk of developing acute respiratory distress syndrome.

## Introduction

Acute respiratory distress syndrome (ARDS) is a life-threatening condition characterised by an onset of diagnosis within 7 days of an apparent clinical insult, bilateral opacities present on chest X-ray and severity defined as ‘mild’ (PaO_2_/FiO_2_=200–300), ‘moderate’ (PaO_2_/FiO_2_=100–200) or ‘severe’ (PaO_2_/FiO_2_<100).^1^ Current treatment strategies are preventative with no therapeutic intervention proven to prevent the devastating effects of ARDS.^2^

Podoplanin is a highly glycosylated cell surface protein expressed on alveolar epithelial type I cells and kidney podocytes, and is upregulated on various immune cell populations, including macrophages, during inflammation.^3^ Its endogenous ligand, C-type lectin-like 2 (CLEC-2), is an immunoreceptor tyrosine-based activation motif-containing cell surface receptor, predominantly expressed on platelets.^4^

Alveolar macrophages (AMs) play important roles in ARDS, serving as innate cellular defences clearing microbes and initiating signals to recruit neutrophils and in regulating resolution of the ongoing inflammatory response.^5 6^ In an unchallenged mouse, the predominant AM population can be identified as CD11c^high^CD11b^neg/low^ using flow cytometry; however, during acute lipopolysaccharide (LPS)-induced lung inflammation, an inflammatory population immerges in the alveoli that is CD11c^high^CD11b^pos^.^7 8^ Our previous data suggest that podoplanin expressed only on these CD11c^high^CD11b^pos^ inflammatory alveolar macrophages (iAMs) may interact with platelet-expressed CLEC-2 to regulate the local inflammatory response.^8^ Therefore, our aim here was to investigate whether therapeutic targeting of the CLEC-2–podoplanin pathway is beneficial during a mouse model of ARDS.

We demonstrate that administration of a crosslinking/activating anti-podoplanin antibody (α-PDPN) in vivo, at the peak of the acute cytokine and chemokine response, significantly reduces lung function decline, with a concomitant reduction in alveolar neutrophilia during intratracheal (IT)-LPS. Furthermore, we show that appropriate podoplanin expression is required to regulate iAM cytokine and chemokine secretion in vitro, suggestive of a potential mechanism for the beneficial effect observed in vivo.

## Methods

### Mice

All mice were maintained in individually ventilated cages at the Biomedical Service Unit (BMSU), University of Birmingham, UK. IT procedures were performed in accordance with UK laws (Animal (Scientific Procedures) Act 1986) with approval of local ethics committee and UK Home Office approval (PPL 40/3741). *Pdpn*^fl/fl^VAV1cre^+^ and floxed-only controls have been described previously.^8^ Wildtype C57Bl/6 mice were purchased from Harlan Laboratories (Oxford, UK).

### Intratracheal LPS

IT instillations of 40 µg LPS (*Escherichia coli* O111:B4; InvivoGen, France) and MouseOx Plus (Starr Life Sciences, USA) were performed as previously described, with sequential MouseOX Plus and body weight measurements taken at 1, 2, 3, 4, 6 and 9 days after IT-LPS.^9^ Using a second cohort of mice, bronchoalveolar lavage (BAL) fluid was extracted post mortem, 48 hours after IT-LPS, to measure alveolar neutrophilia, total protein and local cytokine/chemokine expression, with lung wet:dry weight ratios analysed in a third cohort of mice also taken 48 hours after IT-LPS, as previously described.^8^ Finally, a fourth cohort of mice was assessed 48 hours after IT-LPS for histological assessment by H&E staining. Lungs were inflated post mortem with optimum cutting temperature compound (Tissue-Tek, The Netherlands) in phosphate-buffered saline (PBS) and removed en bloc, frozen over dry ice and sections processed as previously described.^8^

α-PDPN (clone 8.1.1) (100 µg per mouse) was administered via intravenous injection at the peak of local cytokine and chemokine expression, 6 hours after IT instillations,^10^ and compared with PBS-treated mice. Treatments were blinded in non-randomised mice until after data analysis. Wildtype C57Bl/6 male mice, aged 10–14 weeks, were used.

### In vitro alveolar macrophage culture

Alveolar macrophages were isolated post mortem via the trachea in 10× 1 mL PBS BAL washes from male or female *Pdpn*^fl/fl^VAV1cre^+^ and floxed-only control mice, aged 8–17 weeks, as previously described.^11^ Cells were cultured for 24 hours either untreated or treated with 0.1 µg/mL LPS in the presence or absence of an α-PDPN (clone 8.1.1) (10 µg/mL). Secreted cytokines and chemokines were measured in the supernatant by Fluorokine MAP Multiplex (R&D Systems, Abingdon, UK). Cells were harvested in ice-cold 5 mM EDTA in PBS using a 25 cm cell lifter for flow cytometry analysis.

### Statistical analysis

All parameters were analysed using Prism 7 (GraphPad Software, USA). Arterial oxygen (O_2_) saturation, activity and weight change were analysed by two-way repeated-measures analysis of variance (ANOVA), with Sidak’s post-test comparisons. For all other data, normality was confirmed using a Shapiro-Wilk test and significance assessed by one-way ANOVA with Tukey’s multiple comparisons test or Student’s t-test as indicated in the figure legends along with n numbers. Data are presented as box–whisker plots with mean displayed and the range of minimum to maximum data points.

## Results

### Therapeutic administration of α-PDPN reduces neutrophil infiltration and ameliorates lung function decline during IT-LPS

**Figure 1:**
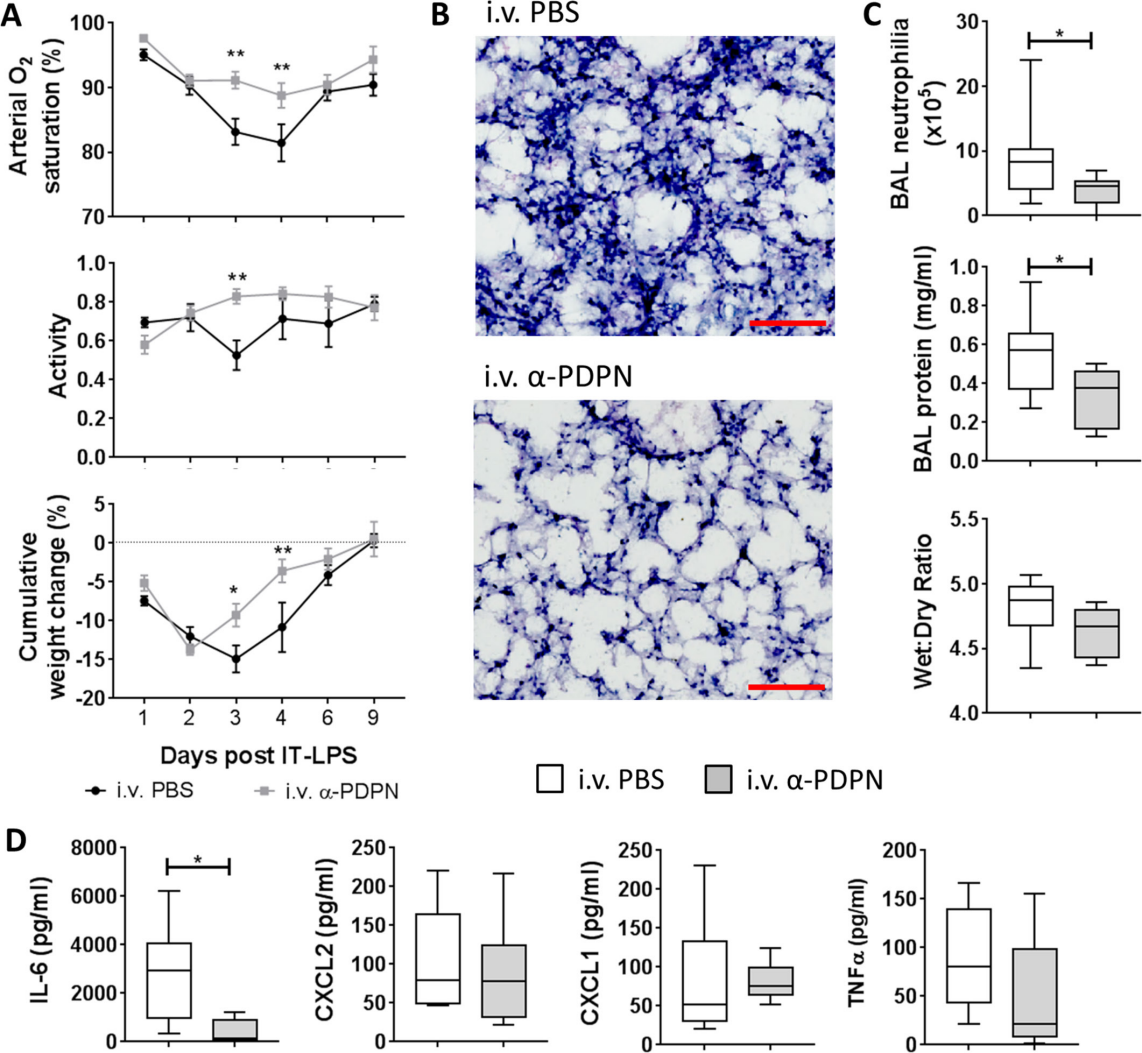
Therapeutic administration of an anti-podoplanin antibody (α-PDPN) is beneficial during a mouse model of acute respiratory distress syndrome. (A) Sao_2_ (P<0.001) and activity (P=0.043) were measured by MouseOx Plus, as well as cumulative weight change of wildtype mice (P=0.007) given intravenous phosphate-buffered saline (PBS) or α-PDPN 6 hours after intratracheal (IT) instillation of lipopolysaccharide (LPS). Mice were monitored for 9 days after IT-LPS (intravenous PBS n=8, intravenous α-PDPN n=7; mean±SEM). Two-way repeated-measures analysis of variance was performed with Sidak’s multiple comparisons test. (B) H&E staining of lungs from wildtype mice given α-PDPN 6 hours after IT instillation of LPS compared with controls (n=4). Bar, 100 µm. (C) Bronchoalveolar lavage (BAL) neutrophilia, total BAL protein and lung wet:dry weight ratio were measured 48 hours after IT-LPS in wildtype mice given intravenous PBS or α-PDPN 6 hours after IT instillation of LPS (BAL neutrophilia and protein n=10; wet:dry ratio n=6). Student’s t-tests were performed. (D) Expression of cytokines/chemokines in BAL were measured 48 hours after IT-LPS in wildtype mice given intravenous PBS or α-PDPN 6 hours after IT instillation of LPS (n=6). Student’s t-tests were performed. *P<0.05; **P<0.01. IL-6, interleukin-6; TNFα, tumour necrosis factor alpha.

Our previous data suggest that the platelet–CLEC-2–podoplanin signalling pathway regulates the immune response during IT-LPS in mice.^8^ Therefore, we wanted to investigate the therapeutic potential of targeting this pathway in vivo. C57Bl/6 wildtype mice were treated 6 hours after administration of IT-LPS with an α-PDPN, which induces crosslinking/activation of podoplanin (α-PDPN; clone 8.1.1, 100 µg per mouse^12 13^). α-PDPN-treated mice demonstrated a significant reduction in arterial O_2_ saturation decline after IT-LPS compared with PBS-treated controls (P<0.001) (figure 1A). No significant change in pulse distention was observed, indicating that the reduction in O_2_ saturation was not due to reduced blood flow (P=0.516; data not shown).^14^ This was accompanied by significant improvement in animal recovery as assessed by increased activity (P=0.043) and weight gain (P=0.007) in α-PDPN-treated mice (figure 1A).

Histological assessment of lung tissue at peak neutrophilia (48 hours after IT-LPS) demonstrated reduced neutrophil infiltration in α-PDPN-treated animals (figure 1B). This was quantified by analysing BAL neutrophilia, which revealed a significant reduction in mice treated with α-PDPN (figure 1C). Total BAL protein 48 hours after IT-LPS was also reduced in α-PDPN-treated mice compared with PBS-treated controls (figure 1C). However, no change in lung wet:dry weight ratio was observed 48 hours after IT-LPS in animals treated with α-PDPN compared with controls (figure 1C).

The level of the acute inflammatory cytokine, interleukin (IL)-6, in BAL fluid was also significantly reduced in α-PDPN-treated mice 48 hours after IT-LPS (figure 1D), further demonstrating the anti-inflammatory effect of α-PDPN therapy during this mouse model.

Taken together, these data confirm the importance of the CLEC-2–podoplanin pathway during IT-LPS and demonstrate the beneficial effect of targeting podoplanin during IT-LPS in mice.

### Podoplanin expression on in vitro-generated inflammatory alveolar macrophages regulates their cytokine/chemokine release

Having observed a beneficial effect of crosslinking/activating podoplanin during IT-LPS, we next considered the possible mechanism for this effect. Our previous data suggest that CLEC-2 on platelets regulates the immune response during IT-LPS via podoplanin specifically expressed on iAMs.^8^ Therefore, murine AMs were isolated following BAL washes and cultured in vitro for 24 hours to generate an inflammatory phenotype, as previously described.^11^ Culture-induced iAMs were identified as F4/80^+^CD11c^+^CD11b^+^ cells using flow cytometry (online supplementary figure 1A). Although variable, the percentage of iAMs present after 24 hours of culture did not alter significantly between conditions (P=0.113; online supplementary figure 1B).

10.1136/bmjresp-2017-000257.supp1 Supplementary file 1

**Figure 2:**
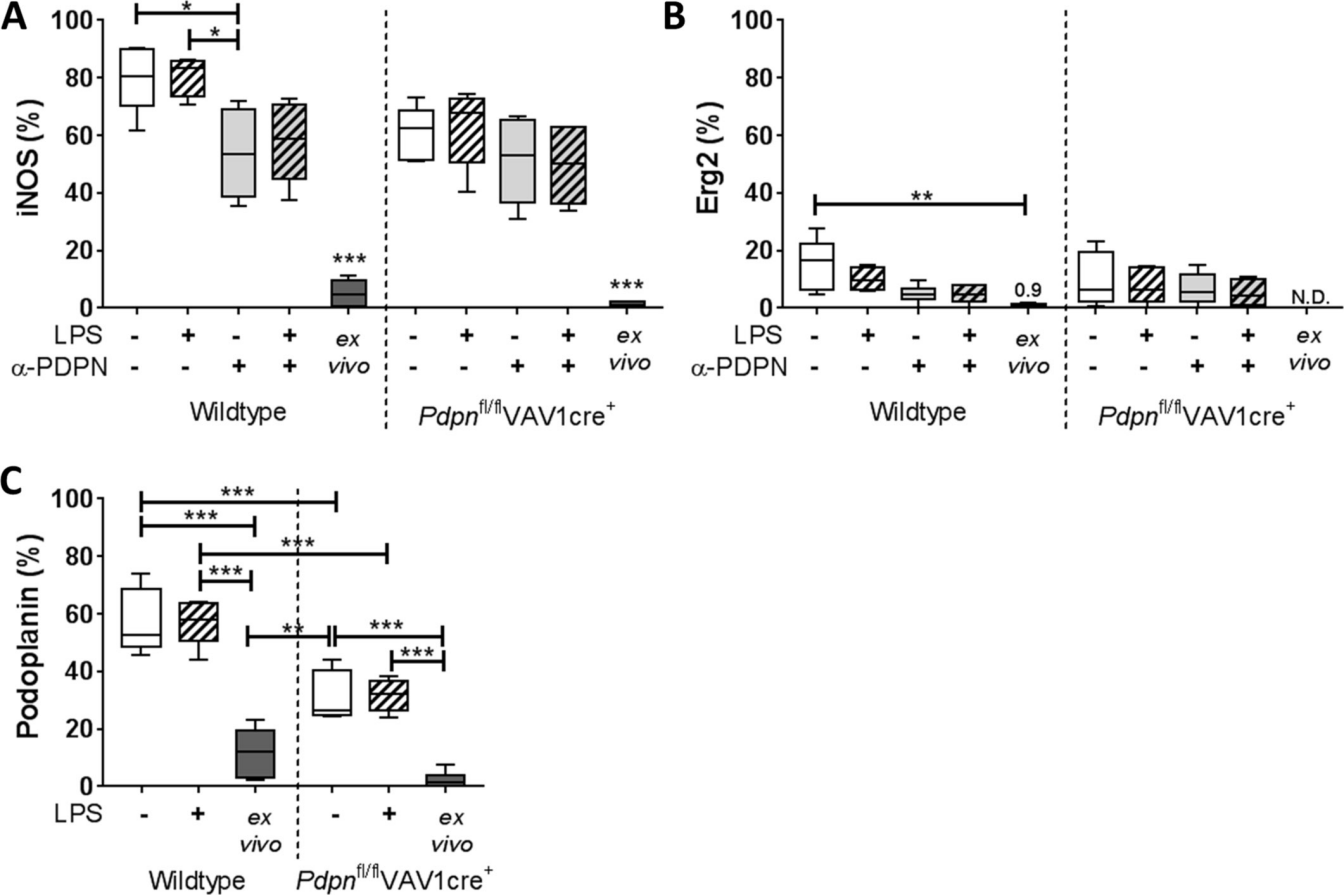
Inducible nitric oxide synthase (iNOS), early growth response protein 2 (Erg2) and podoplanin expression on in vitro-generated inflammatory alveolar macrophages. Bronchoalveolar lavage (BAL) alveolar macrophages (AMs) isolated from *Pdpn*^fl/fl^VAV1cre^+^ or floxed-only controls (wildtype) were cultured for 24 hours and treated with or without lipopolysaccharide (LPS), in the presence or absence of anti-podoplanin antibody (α-PDPN). (A) Cell surface expression of iNOS, (B) Erg2 and (C) podoplanin assessed by flow cytometry and compared with AMs isolated without culturing (ex vivo). One-way analysis of variance was performed on each parameter with Tukey’s multiple comparisons test (n=5–6).  *P<0.05; **P<0.01; ***P<0.001. N.D., not determined.

The inflammatory phenotype of the iAMs was first confirmed by high expression of the M1 marker, inducible nitric oxide synthase (iNOS),^15^ compared with low expression of the M2 marker, early growth response protein 2 (Erg2)^16^ (figure 2A and B, respectively; gating strategy is indicated in online supplementary figure 1A). In untreated conditions and in line with our in vivo data,^8^ podoplanin is also expressed on culture-generated iAMs (57.5%±11.2%) compared with AMs isolated straight ex vivo (11.7%±8.4%) derived from wildtype mice (figure 2C).

Constitutive deletion of podoplanin is lethal in murine models^17^; therefore, to investigate the function of podoplanin on these macrophages, we used a haematopoietic-specific podoplanin-deficient mouse strain (*Pdpn*^fl/fl^VAV1cre^+^^8^). As expected, significantly lower podoplanin expression is observed on cultured iAMs isolated from *Pdpn*^fl/fl^VAV1cre^+^ mice compared with wildtype controls (31.2%±8.7%) (figure 2C).

The addition of LPS (0.1 µg/mL) to cultures for 24 hours did not significantly alter expression of iNOS, Erg2 or podoplanin on iAMs derived from *Pdpn*^fl/fl^VAV1cre^+^ or wildtype controls (figure 2A,B). Cultured AMs were also treated with the α-PDPN antibody for 24 hours to induce crosslinking/activation of podoplanin in the presence or absence of LPS (α-PDPN; clone 8.1.1, 10 µg/mL^12 13^). A significant reduction in iNOS expression was observed on iAMs in α-PDPN-treated cells derived from wildtype mice (P=0.023), which remained consistent in the presence of LPS (figure 1A). Conversely, α-PDPN treatment did not alter iNOS expression in iAMs derived from *Pdpn*^fl/fl^VAV1cre^+^ mice (P=0.954).

**Figure 3:**
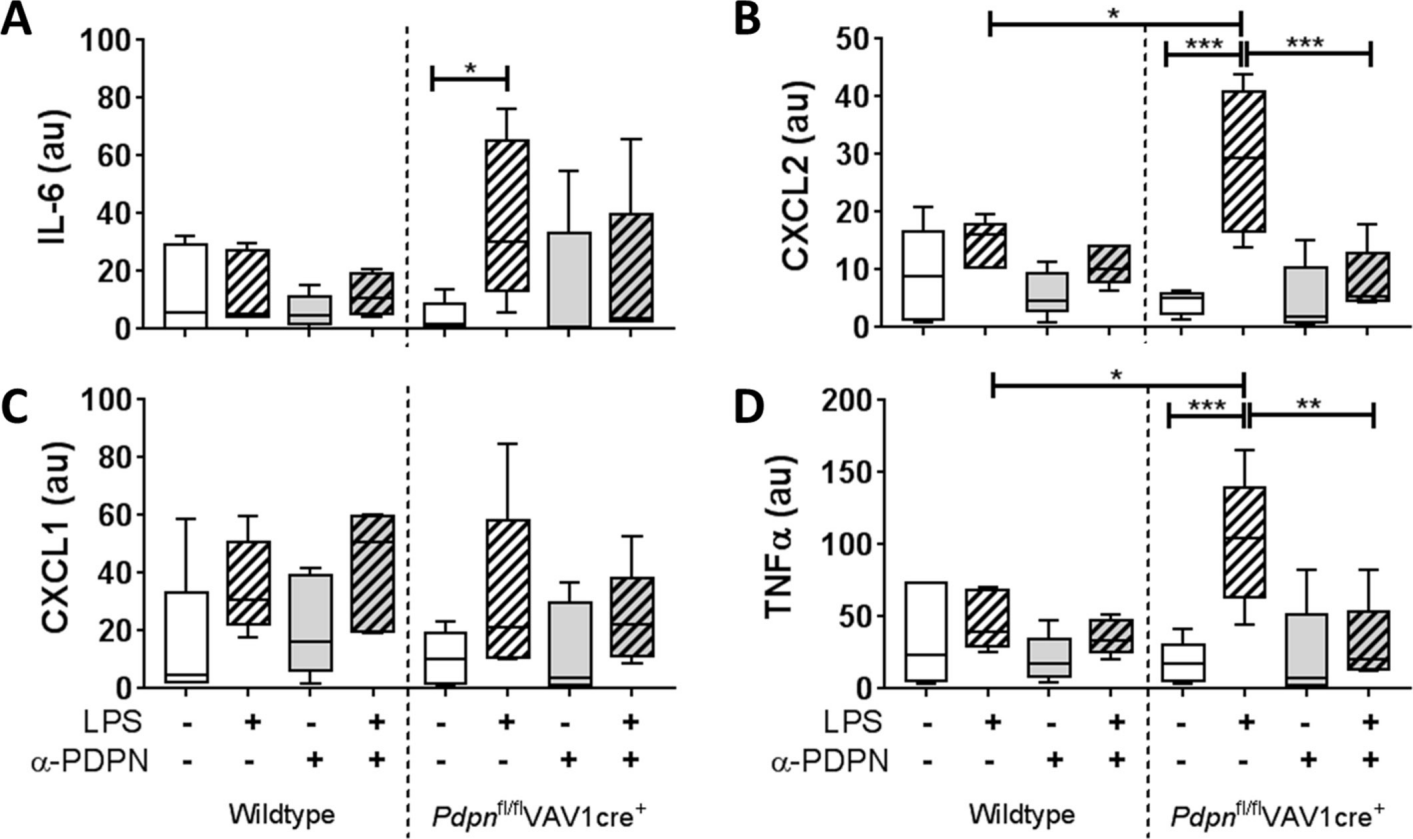
Podoplanin expression on inflammatory alveolar macrophages (iAMs) regulates their immune function in vitro. Expression of (A) interleukin-6 (IL-6), (B) CXCL2, (C) CXCL1 and (D) tumour necrosis factor alpha (TNFα) in the media of in vitro-generated iAMs measured after 24 hours of culture, treated with or without LPS, in the presence or absence of anti-podoplanin antibody (α-PDPN) (n=5). Data were normalised to the percentage of iAMs present in the culture and presented in arbitrary units (au). One-way analysis of variance was performed with Tukey’s multiple comparisons test. *P<0.05; **P<0.01; ***P<0.001.

The functional relevance of podoplanin expression and/or α-PDPN treatment was assessed by measuring inflammatory cytokines/chemokines in the media of 24hour-cultured AMs. All data are presented normalised to the percentage of iAMs present in the culture. Expression of IL-6, chemokine (C-X-C motif) ligand (CXCL)2, CXCL1 and tumour necrosis factor alpha (TNFα) were not significantly altered in iAMs derived from wildtype mice when cultured with LPS compared with LPS-free conditions (figure 3). In addition, α-PDPN treatment did not significantly affect these levels. Conversely, expression of IL-6, CXCL2 and TNFα were significantly increased in cultured cells derived from *Pdpn*^fl/fl^VAV1cre^+^ mice when cultured in the presence of LPS compared with LPS-free conditions (19.1-fold, 7.4-fold and 8.1-fold, respectively) (figure 3A,B,D). Addition of α-PDPN significantly reduced these levels back to LPS-free levels, suggesting that α-PDPN is only effective in hyper-responsive environments even when podoplanin expression is reduced. Furthermore, LPS-treated *Pdpn*^fl/fl^VAV1cre^+^-derived iAMs express significantly higher levels of CXCL2 and TNFα compared with LPS-treated wildtype iAMs (2.0-fold and 2.5-fold, respectively) (figure 3B and D, respectively).

Together, these data suggest that podoplanin expressed on iAMs regulates their release of proinflammatory cytokines and chemokines in culture.

## Discussion

The principal findings of this study are that (1) targeting podoplanin in vivo limits acute lung inflammation, improving animal welfare during IT-LPS, and (2) podoplanin expressed on in vitro-generated inflammatory alveolar macrophages regulates their cytokine/chemokine secretion.

Our previous work suggested that the interaction between platelet–CLEC-2 and podoplanin expressed on iAMs limits excessive lung inflammation during IT-LPS in mice.^8^ Specifically, the data ruled out the contribution of other lung resident podoplanin-expressing haematopoietic cells, alveolar type I cells and lymphatic cells by the use of conditional knockout mouse models in vivo. Together with the data presented here, our findings suggest that expression and/or activation of podoplanin on iAMs is required for appropriate cytokine/chemokine release from these cells, which influences neutrophil recruitment and, in turn, lung function during IT-LPS. However, there remains no direct evidence for this or for the direct platelet–CLEC-2-mediated activation of podoplanin on iAMs. Delineating when and how this happens during IT-LPS will be the focus of future studies.

An increase in BAL neutrophilia and total protein was observed in α-PDPN-treated animals during IT-LPS but not lung wet:dry lung weight. This is indicative of the α-PDPN treatment reducing lung inflammation without affecting the increase in lung permeability observed following IT-LPS. These results were similar to those observed using multiple conditional CLEC-2/podoplanin-deficient mouse strains 48 hours after IT-LPS,^8^ indicative of this pathway’s major role in protecting against excessive pulmonary inflammation rather than affecting barrier function in this model.

There are limitations to this study. First is the assumption that the iAMs generated by in vitro culturing relate directly to those observed in the alveolar space in vivo following IT-LPS. Using bone marrow chimeras combined with intranasal LPS administration, the origin of iAMs has been described to be from both tissue/alveolar resident and blood precursors.^7^ Therefore, we chose to generate iAMs directly from alveolar resident AMs as previously reported.^11^ However, the composition of AM subpopulations in vivo is diverse and dynamic, especially during inflammation,^18^ and thus whether the culture-generated iAMs truly represent those present during IT-LPS remains speculative. It will therefore be important in future studies to fully characterise these podoplanin-expressing iAMs beyond the parameters tested in this study and also use bloodborne AM precursors to investigate the role of podoplanin on this subpopulation of cells.

Second, we observed only a reduction in podoplanin expression in *Pdpn*^fl/fl^VAV1cre^+^-derived iAMs, possibly due to the mixed progenitor population, which contributes to AMs in adult mice as previously reported.^19^ Ideally, a complete knockout of podoplanin is required to fully assess its function; however, global deletion of podoplanin is embryonic lethal in mouse models.^17^ Given that an approximately 50% reduction in protein expression still had a significant effect on CXCL2 and TNFα expression is supportive of the importance of podoplanin in this in vitro system.

Finally, we only used one in vivo model of ARDS to assess the therapeutic targeting of podoplanin. IT-LPS predominantly reproduces the acute neutrophilic inflammatory responses that occur during ARDS in patients. Other aspects such as severe endothelial and epithelial injury as well as repair with fibrosis are less apparent or absent in this model.^20^ It will therefore be of paramount importance to test the α-PDPN in other models of ARDS.

In conclusion, this study demonstrates that therapeutic administration of an α-PDPN is beneficial during a mouse model of ARDS in vivo. This may be via activation of podoplanin expressed on iAMs, altering local cytokine/chemokine expression, which in turn regulates neutrophil recruitment. Targeting podoplanin may therefore represent a novel avenue for future therapeutics in patients at risk of developing ARDS.
