# Supplementary material for: Effect of anti-podoplanin antibody administration during lipopolysaccharide-induced lung injury in mice
Source: BMJ Open Respir Res. 2017 Nov 8;4(1):e000257. doi: 10.1136/bmjresp-2017-000257 (PMC5687585; doi:10.1136/bmjresp-2017-000257)
Supplement: Supplementary file 1 [file bmjresp-2017-000257supp001.pdf]

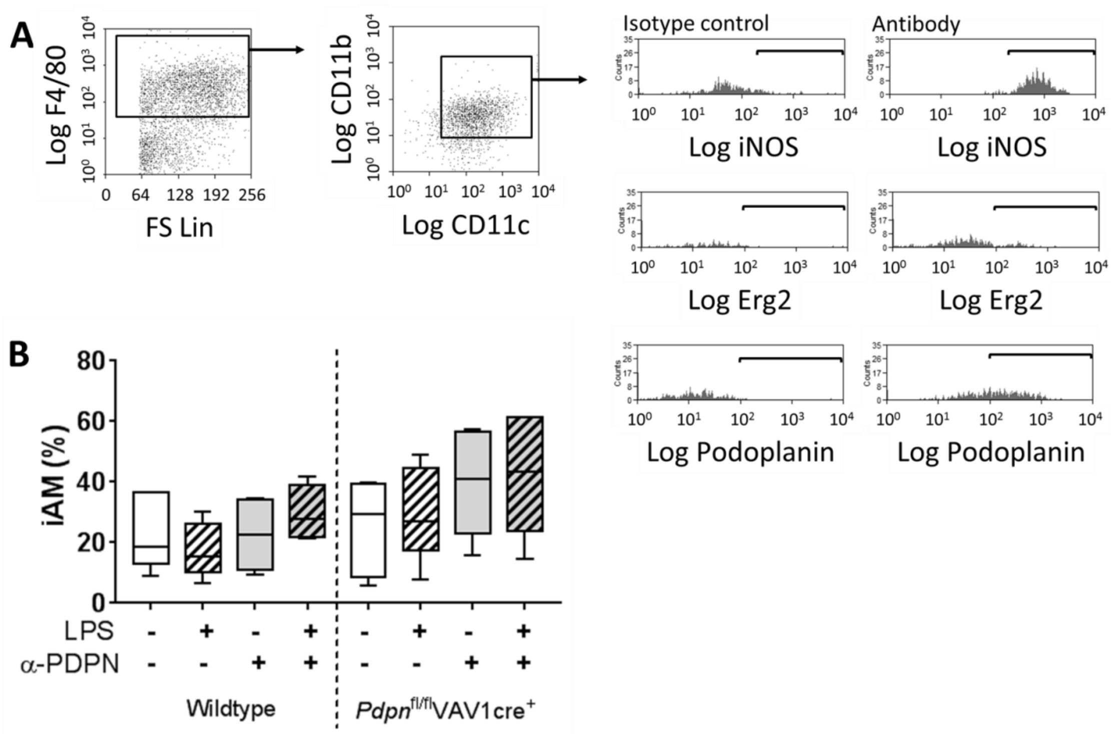

Supplementary Figure 1. *In vitro* generation of inflammatory alveolar macrophages.

(A) Representative histograms for the identification of cultured inflammatory alveolar macrophages (iAMs) derived from bronchoalveolar lavage (BAL) alveolar macrophages (AM) isolated from *Pdpn*$^{fl/fl}$ VAV1cre^{+} or floxed-only controls (wildtype) and cultured for 24 hours. Inflammatory AMs are identified using flow cytometry based on their expression of F4/80, CD11c and CD11b. Expression of inducible nitric oxide synthase (iNOS), early growth response protein 2 (Egr2) and podoplanin were analysed by setting the gate at $\leq$1% using the cognate isotype control as shown. (B) The percentage of iAMs present after 24 hours of culture do not alter significantly between conditions (One-way ANOVA; p=0.113) (n=5-6).
